# The Predictive Value of the Tumor‐Stroma Ratio for Neoadjuvant Endocrine Therapy in Hormone Receptor‐Positive Breast Cancer

**DOI:** 10.1002/ijc.70490

**Published:** 2026-04-10

**Authors:** Layla Andour, Sophie C. Hagenaars, Anne Florine de Groot, Elly M. M. Krol‐Warmerdam, Judith R. Kroep, Hans Marten Hazelbag, Gerrit‐Jan Liefers, Marieke E. Straver, Wilma E. Mesker

**Affiliations:** ^1^ Department of Surgery Leiden University Medical Center Leiden the Netherlands; ^2^ Department of Medical Oncology Leiden University Medical Center Leiden the Netherlands; ^3^ Department of Pathology Haaglanden Medical Center The Hague the Netherlands; ^4^ Department of Surgery Medical Center Haaglanden The Hague the Netherlands

**Keywords:** breast cancer, endocrine therapy, magnetic imaging resonance, neoadjuvant systemic therapy, tumor‐stroma ratio

## Abstract

Approximately 75% of breast cancers are hormone receptor‐positive (HR+). Endocrine therapy may be administered to improve survival. Neoadjuvant therapy may enable breast‐ and axilla‐conserving surgery, but identifying who will benefit remains challenging. This study evaluates the predictive value of the tumor‐stroma ratio (TSR) for response to neoadjuvant endocrine therapy (NET) and examines the accuracy of MRI in predicting (near) pathological complete response (pCR). Women diagnosed between 2014 and 2024 with invasive HR+, human epidermal growth factor 2‐negative breast cancer who received NET in two Dutch hospitals were included. A total of 208 women (215 tumors) were included, of whom 81 (37.7%) had a stroma‐low tumor and 134 (62.3%) stroma‐high. Almost 1 out of 4 stroma‐low cases achieved (near) pCR after NET. In both univariable (OR 2.85, 95% CI 1.32–6.15, *p* = 0.008) and multivariable analysis (OR 3.70, 95% CI 1.54–8.90, *p* = 0.003), stroma‐low tumors showed an increased likelihood of achieving (near) pCR compared to stroma‐high tumors. In node‐positive patients (*n* = 117), univariable analysis presented higher odds of pCR in the axilla after NET in the stroma‐low group (OR 4.41, 95% CI 1.08–18.08, *p* = 0.039), although this was not significant in multivariable analysis (*p* = 0.060). The MRI correctly reported (near) pCR in 12 (75%) stroma‐low cases and 8 (35%) stroma‐high. Stroma‐low patients significantly more often achieved (near) pCR of the tumor and axilla after NET compared to stroma‐high patients. The accuracy of MRI to predict (near) pCR was higher in the stroma‐low group compared to the stroma‐high group.

## Abstract (highlights)

What's new?

Neoadjuvant endocrine therapy (NET) can help prevent metastases and improve survival in patients with hormone receptor‐positive (HR+) breast cancer. Identifying which HR+ patients will benefit from NET, however, remains challenging. Here, in a patient cohort in the Netherlands, the authors evaluated tumor‐stroma ratio (TSR) and magnetic resonance imaging (MRI) accuracy for predicting NET response. Stroma‐low tumors were significantly more likely to achieve near‐complete pathological responses in breast and axilla compared to stroma‐high tumors. MRI more accurately predicted response in stroma‐low cases. The findings highlight the utility of TSR in predicting NET response and aiding treatment planning in HR+ breast cancer.

## Graphical abstract

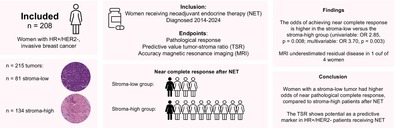

**Abbreviations**
*κ*
: Cohen's kappa
*χ* ^2^
: Pearson chi‐square
ALND: axillary lymph node dissection
BCS: breast‐conserving surgery
BOOG: Dutch Breast Cancer Research Group
CI: confidence interval
DCIS: ductal carcinoma in situ
ER: estrogen receptor
H&E: hematoxylin and eosin
HER2: human epidermal growth factor receptor 2
HMC: Medical Center Haaglanden
HR: hormone receptor
IHC: immunohistochemical
IKNL: The Netherlands Comprehensive Cancer Organization
LUMC: Leiden University Medical Center
MP: Miller‐Payne
MRI: magnetic resonance imaging
MST: mastectomy
N: lymph node
NACT: neoadjuvant chemotherapy
NEOLBC: Tailoring NEOadjuvant Therapy in HR+/HER2−, Luminal Breast Cancer
NET: neoadjuvant endocrine therapy
OR: odds ratio
pCR: pathological complete response
PR: progesterone receptor
RCB: residual cancer burden
rCR: radiological complete response
RCT: randomized controlled trial
RECIST: response evaluation criteria in solid tumors
T: tumor
TME: tumor microenvironment
TSR: tumor‐stroma ratio

## 1 Introduction

Hormone receptor‐positive (HR+) breast cancer is the most frequently occurring (75%) subtype, characterized by positive estrogen receptor (ER) and/or progesterone receptor (PR) expression [1]. The American Society of Clinical Oncology advises endocrine therapy in case the tumor is > 0.5 cm, whereas the European Society for Medical Oncology recommends it in all HR+ cases [2, 3].

Endocrine therapy, in addition to surgery, is administered to prevent metastases and improve survival [4, 5]. Advantages of therapy in the neoadjuvant setting are downstaging of both the breast and axilla, and evaluating the efficacy of therapy. This means that women who initially are not eligible to undergo breast conserving surgery (BCS) now have a possibility to be considered for BCS instead of mastectomy (MST). Furthermore, downstaging of the axilla may result in less invasive procedures and prevent an axillary lymph node dissection (ALND) instead.

It is still a challenge to identify who gains advantage from neoadjuvant therapy, especially since randomized controlled trials (RCTs) on primary surgery versus NET are lacking. According to the Dutch guidelines, the therapy choice in women with HR+ and human epidermal growth factor receptor 2‐negative (HER2−) breast cancer generally depends on tumor size, grade, and involvement of lymph nodes, with overlapping indications for both NACT and NET [6]. Tools such as PREDICT, Oncotype DX, and MammaPrint are available in an attempt to predict which patient will benefit from additional systemic therapy, next to surgery, but are mainly focused on chemotherapy [7, 8, 9]. Furthermore, clinical examination and imaging, such as magnetic resonance imaging (MRI) and ultrasounds, are not always accurate in evaluating the response to neoadjuvant therapy, especially after NET, due to the scattered and irregular pattern of tumor regression (cookie crumble effect) that occurs [10, 11, 12]. Therefore, there is a clinical need to better understand who might profit from neoadjuvant endocrine therapy, to prevent under‐ and overtreatment.

One potential marker that could help stratify patients according to their predicted response to neoadjuvant therapy is the tumor‐stroma ratio (TSR), which not solely focuses on tumor cells but involves the tumor microenvironment (TME) [13]. High stromal content has shown to be correlated with worse survival outcomes in breast cancer patients [14, 15, 16, 17]. An earlier study by our research group also assessed the predictive value of the TSR for NACT and found that patients with stroma‐high tumors have worse treatment response rates compared to those with stroma‐low tumors [18]. Studies on the predictive value of the TSR in breast cancer patients receiving NET are still lacking.

The TSR might aid in better selecting women who benefit from neoadjuvant therapy, potentially contributing to decision‐making in clinical practice. Moreover, the TSR might complement and strengthen the accuracy of the MRI in assessing response after NET, thereby improving the prediction and assessment of the tumor size and facilitating more precise preoperative planning. In this study, the predictive value of the TSR and MRI for the response to neoadjuvant endocrine therapy in HR+/HER2− breast cancer patients will be examined.

## 2 Material and Methods

Patients in this retrospective, observational study were either participants of a Dutch Breast Cancer Research Group (BOOG) coordinated phase II trial—called Tailoring NEOadjuvant therapy in HR+/HER2−, Luminal Breast Cancer (NEOLBC) – or received treatment as part of standard clinical care in two hospitals in the Netherlands.

### 2.1 Study Population

#### 2.1.1 NEOLBC Trial

Within the NEOLBC trial (NCT03283384), postmenopausal women with ER+, HER2‐negative, stage II/III breast cancer were included from June 2018 to March 2021 [19]. All women received 2 weeks of letrozole after which a new biopsy was taken. Patients with a Ki67 < 1% continued to receive hormonal therapy, whereas all other patients were randomized to receive other treatment modalities. Women who received NET were included in this study, and those in other treatment groups were excluded.

#### 2.1.2 Clinical Care

Women who received NET at the Leiden University Medical Center (LUMC) or Medical Center Haaglanden (HMC) in the Netherlands, and who were diagnosed between 2014 and 2024 with HR+/HER2− breast cancer, were included in this study. All women received NET according to the Dutch breast cancer guideline, which recommends aromatase inhibitors such as letrozole as preferred therapy or tamoxifen, based on characteristics including menopausal status and clinical judgment [6]. The Netherlands Comprehensive Cancer Organization (IKNL) aided in providing the list of patients who received neoadjuvant endocrine therapy in the hospitals. Exclusion criteria consisted of other neoadjuvant systemic therapy regimens, ductal carcinoma in situ (DCIS) in the biopsy with no invasive component, and the absence of data on pathological response rates.

### 2.2 Tumor‐Stroma Ratio

**FIGURE 1:**
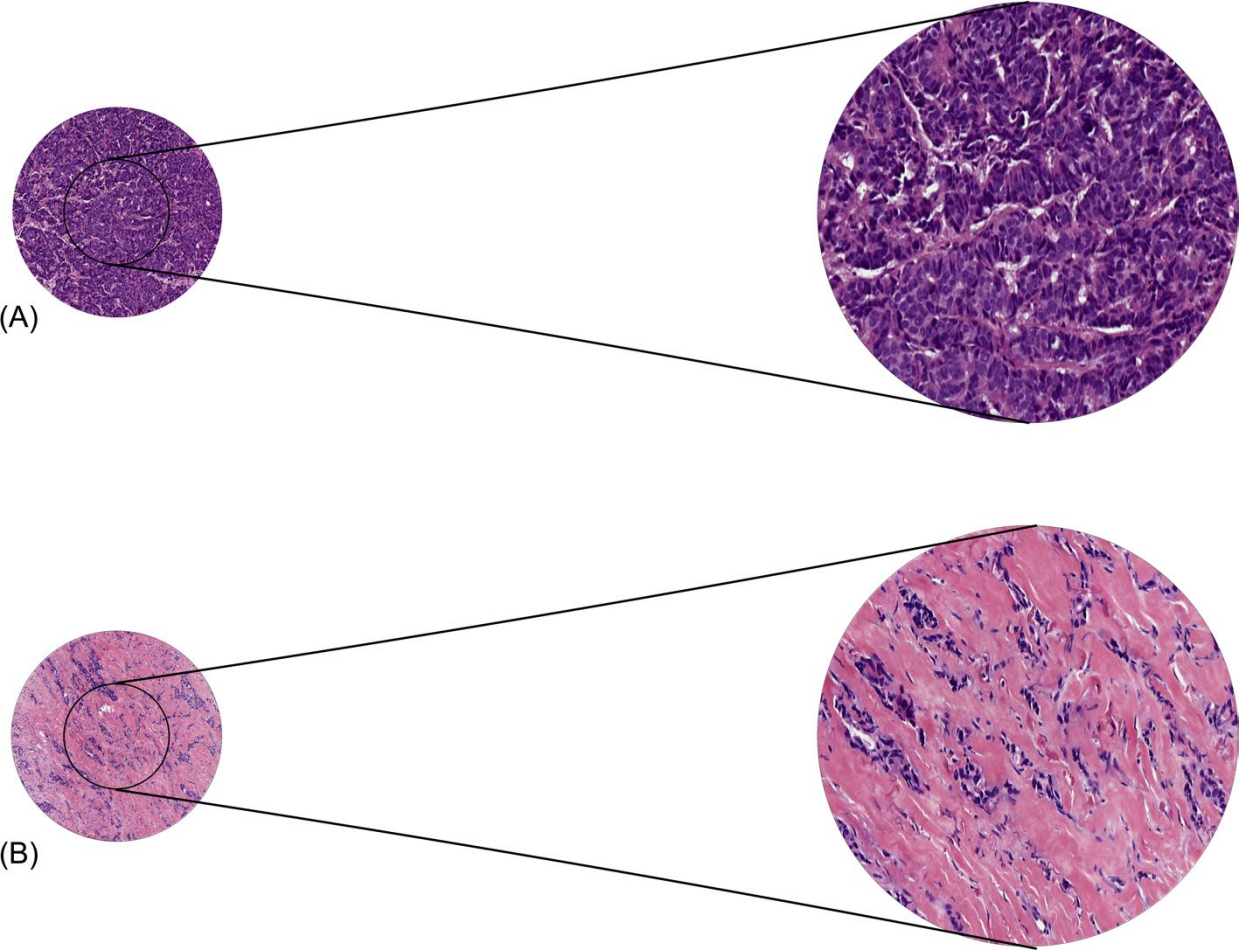
(A) Stroma‐low tumor (10% stroma), (B) stroma‐high tumor (70% stroma).

Scoring of the TSR was performed on the diagnostic biopsies of untreated patients. The process of scoring was according to protocol on hematoxylin and eosin (H&E)‐stained slides by visually eyeballing the percentage of stroma, compared to tumor cells [20]. The TSR was scored in the invasive components and not in areas containing in situ carcinoma. Stromal content is evaluated in increments of 10%, with low stroma defined as ≤ 50% presence of stroma (Figure 1A), and > 50% stroma (Figure 1B) considered as stroma‐high as previously determined [20, 21]. All slides were scored by two researchers (LA, SCH) and a third, experienced researcher in case of discrepancy (WEM). Cases that were difficult to interpret or those without clear pathological response scores were reviewed by specialized breast cancer pathologists (HMH, VTHBMS).

### 2.3 Outcomes

The primary endpoint was the pathological response rate, in association with the TSR. Secondary endpoints consisted of the accuracy of the TSR and MRI‐based radiological response, each in relation to the pathological response.

#### 2.3.1 Definitions

The luminal A‐like subtype was defined as ER+/PR+ (Grades I–II), and the luminal‐B like as ER+/PR+ (Grade III) or ER+/PR− (Grades I–III). ER and/or PR expression was considered positive in case of ≥ 10% expression. Clinically node positive (cN+) status was defined by clinical examination and/or axillary ultrasound, with fine‐needle aspiration performed for lymph nodes with a cortical thickness > 2.3 mm, and core needle biopsy considered in case of inconclusive cytology. Pathological response outcomes were obtained from pathology reports of the resection specimens, which were evaluated by in‐house pathologists specialized in breast pathology. Across the hospitals, different grading classifications were reported in the pathological reports: in some cases, the Miller‐Payne (MP) classification was applied, whereas in others the Residual Cancer Burden (RCB) was used. Therefore, in this study, the categories were divided into no response, partial response (10%–90% tumor rest) and (near) complete response (< 10% tumor rest) to allow comparison across groups. The < 10% threshold is consistent with prior studies using MP Grades 4–5 to indicate minimal or no residual disease, allowing standardized comparisons across response categories [18, 22]. For the assessment of RCB, tumor size was determined using pre‐ and post‐treatment imaging (ultrasound/MRI) and post‐treatment tissue sections. The primary tumor bed was identified macroscopically in the resection specimen, often as fibrosis, after which residual tumor was assessed microscopically by estimating the percentage of viable invasive tumor cells within the defined tumor bed. Tumor stroma (TSR) was assessed only on pre‐treatment biopsies, as stromal content may change during therapy due to fibrosis; all assessments were performed by experienced in‐house breast pathologists.

Response of the axilla was divided into no response (defined as isolated tumor cells and N1–N3) or complete response (pCR). Complete response was defined as no residual tumor cells or in situ carcinoma. Radiological response was assessed on preoperative MRI images by in‐house radiologists and was divided into no response (no response and progression of disease), partial response, and radiological complete response (rCR). Radiological response was mostly assessed according to Response Evaluation Criteria In Solid Tumors (RECIST) criteria, as generally applied in accordance with national guideline recommendations [6].

### 2.4 Data Analysis

For the assessment of the endpoints, analyses were performed in IBM SPSS Statistics version 29. Cohen's kappa (*κ*) was performed to evaluate interobserver agreement. Comparison of patient characteristics between stroma‐low and stroma‐high groups was performed using Mann–Whitney *U* test for continuous variables and Pearson chi‐square (*χ* ^2^) test for categorical variables. Associations between the TSR, radiological, and pathological response of the tumor and axilla were analyzed with the *χ* ^2^ test. Univariable and multivariable logistic regression analyses were performed to evaluate the odds of achieving (near) complete response and to adjust for variables correlated with pathological response, such as age, luminal subtype, morphology, and clinical T‐ and N‐stage. Grade was not included in multivariable analysis, since it is already incorporated in the luminal subtype definition and would have resulted in overlap (collinearity) otherwise. For the axillary response analysis, clinical T‐stage was dichotomized (T1, T2, and T3–T4) due to low cell counts within the response groups and resulting instability in model estimates. In the breast response analysis, T‐stage was retained as a four‐category variable. A *p* value of < 0.05 was considered statistically significant. The reporting of this study is in accordance with the REMARK guidelines [23].

## 3 Results

### 3.1 Patient Characteristics

**FIGURE 2:**
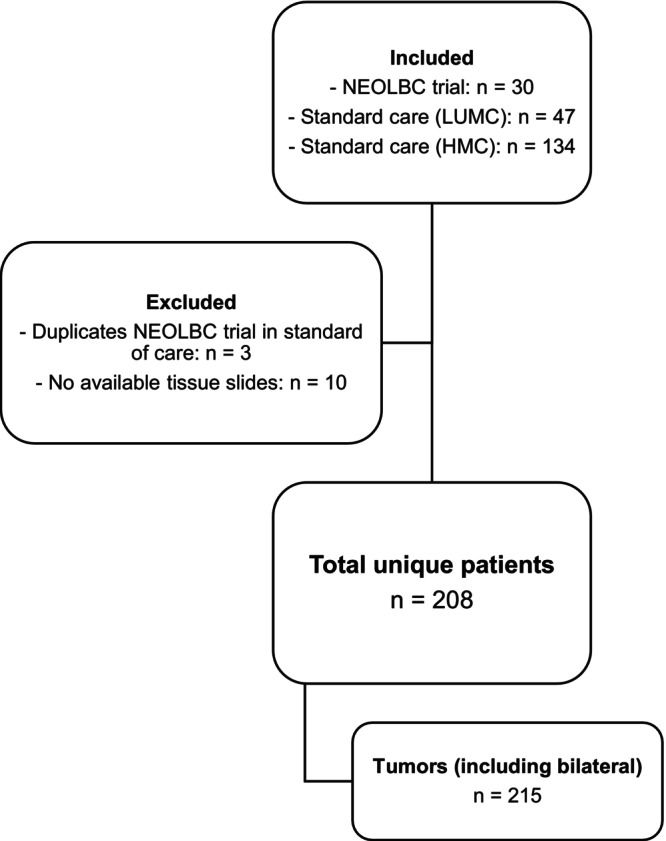
Flowchart of included patients and available tissue slides.

A total of 208 patients with 215 invasive tumors were included in this study (Figure 2). A detailed overview of the characteristics of the patients is presented in Table 1. In case of a multifocal or bilateral tumor, details of the largest primary tumor are described.

**TABLE 1:** Baseline characteristics of included patients.

Characteristics	Patients (*n* = 208, 100%)	Stroma‐low (*n* = 76, 100%)	Stroma‐high (*n* = 132, 100%)	*p*
Median age in years [range]	67.9 [35–89]	67 [35–88]	69.3 [41–89]	0.419
Stroma in first tumor				N/A
Low	76 (36.5)	76 (100.0)	—	
High	132 (63.5)	—	132 (100.0)	
PR‐expression				0.509
PR+	148 (71.2)	52 (68.4)	96 (72.7)	
PR−	60 (28.8)	24 (31.6)	36 (27.3)	
Grade				0.008
I	67 (32.4)	18 (24.0)	49 (37.1)	
II	116 (56.0)	42 (56.0)	74 (56.1)	
III	24 (11.6)	15 (20.0)	9 (6.8)	
Unknown	1	1	0	
Luminal‐subtype				0.127
Luminal A‐like	130 (62.8)	42 (56.0)	88 (66.7)	
Luminal B‐like	77 (37.2)	33 (44.0)	44 (33.3)	
Unknown	1	1	0	
Morphology				0.473
Ductal	151 (73.3)	56 (73.7)	95 (73.1)	
Lobular	48 (23.3)	16 (21.1)	32 (24.6)	
Other	7 (3.4)	4 (5.3)	3 (2.3)	
Unknown	2	0	2	
Clinical T‐stage				0.094
T1	48 (23.1)	11 (14.5)	37 (28.0)	
T2	115 (55.3)	46 (60.5)	69 (52.3)	
T3	24 (11.5)	12 (15.8)	12 (9.1)	
T4	21 (10.1)	7 (9.2)	14 (10.6)	
Clinical N‐stage				0.474
N0	83 (40.9)	29 (39.2)	54 (41.9)	
N1	104 (51.2)	40 (54.1)	64 (49.6)	
N2	8 (3.9)	4 (5.4)	4 (3.1)	
N3	8 (3.9)	1 (1.4)	7 (5.4)	
Unknown	5	2	3	
Clinical stage				0.296
I	13 (6.4)	2 (2.7)	11 (8.6)	
II	138 (68.3)	55 (74.3)	83 (64.8)	
III	44 (21.8)	14 (18.9)	30 (23.4)	
IV	7 (3.5)	3 (4.1)	4 (3.1)	
Unknown	6	2	4	
Bilateral				0.505
No	196 (94.2)	70 (92.1)	126 (95.5)	
Yes (DCIS other side)	5 (2.4)	2 (2.6)	3 (2.3)	
Yes (invasive both sides)	7 (3.4)	4 (5.3)	3 (2.3)	
Type of endocrine therapy				0.147
Letrozole	178 (95.2)	68 (97.1)	110 (94.0)	
Tamoxifen	8 (4.3)	1 (1.4)	7 (6.0)	
Both (switched)	1 (0.5)	1 (1.4)	0 (0.0)	
Unknown	21	6	15	
Surgical technique				0.415
Mastectomy	72 (34.6)	29 (38.2)	43 (32.6)	
Breast conserving	136 (65.4)	47 (61.8)	89 (67.4)	

The median age at diagnosis was 67.9 [35–89] years. All women in the cohort had ER+ breast cancer.

A total of 148 (71.2%) patients had a PR+ tumor and 60 (28.8%) PR− cancer. Luminal A‐like breast cancer was observed in 130 (62.8%) patients and luminal B‐like in 77 (37.2%). Patients with a stroma‐high tumor more frequently had a Grade I tumor (*n* = 49, 37.1%), compared to those with stroma‐low breast cancer (*n* = 18, 24.0%; *p* = 0.008). There was no difference regarding luminal A‐ or B‐like subtypes across the stroma‐low and stroma‐high groups (*p* = 0.127). In the entire cohort, the median duration of NET therapy was 8.5 months [0.7–15.2]. No difference in duration was observed between the stroma‐low and stroma‐high groups (*p* = 0.765). Furthermore, there was no difference regarding the type of NET across the stroma‐low and stroma‐high groups (Table 1, *p* = 0.147).

### 3.2 Tumor Stroma Ratio and Pathological Response

#### 3.2.1 Response of the Breast After Neoadjuvant Therapy

The TSR of all 215 invasive tumors was assessed to evaluate breast tumor response after NET (Table 2). A stroma‐low tumor was observed in 81 (37.7%) cases, and stroma‐high in 134 (62.3%). Cohen's *κ* indicated substantial interobserver agreement (0.703).

**TABLE 2:** Pathological outcomes of the breast after neoadjuvant endocrine therapy.

Pathological outcomes	Invasive tumors (*n* = 215, 100%)	Stroma‐low (*n* = 81, 100%)	Stroma‐high (*n* = 134, 100%)	*p*
Pathological T‐stage				0.031
T0	10 (4.7)	7 (8.6)	3 (2.2)	
Tis	3 (1.4)	3 (3.7)	0 (0.0)	
T1	100 (46.5)	33 (40.7)	67 (50.0)	
T2	77 (35.8)	29 (35.8)	48 (35.8)	
T3	22 (10.2)	9 (11.1)	13 (9.7)	
T4	3 (1.4)	0 (0.0)	3 (2.2)	
Pathological response breast				0.022
No	57 (26.5)	20 (24.7)	37 (27.6)	
Partial	126 (58.6)	42 (51.9)	84 (62.7)	
(Near) complete	32 (14.9)	19 (23.5)	13 (9.7)	

**FIGURE 3:**
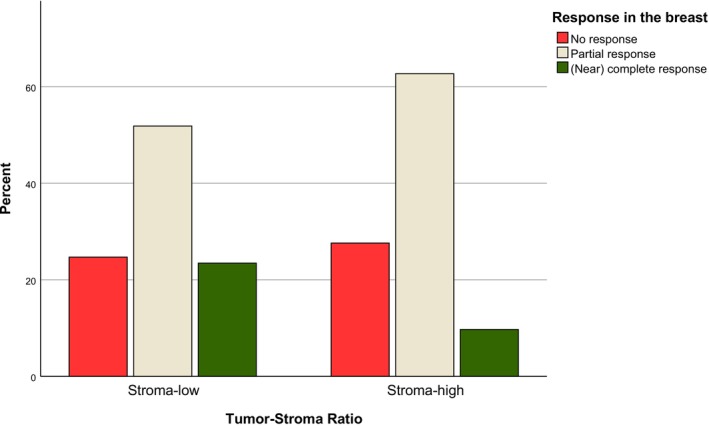
Response in the breast after neoadjuvant endocrine treatment in the stroma‐low and stroma‐high groups.

In 10 (12.3%) women in the stroma‐low group, pCR of the breast was achieved, versus 3 (2.2%) in the stroma‐high group after NET (Table 2). For the *χ* ^2^ analysis, two possible pathological outcomes were used: no or partial response versus (near) pCR. Women with a stroma‐low tumor significantly more often reached (near) pCR (*n* = 19, 23.5%), compared to those with a stroma‐high tumor (*n* = 13, 9.7%, *p* = 0.006, Figure 3).

In univariable logistic regression analysis, a patient with a stroma‐low tumor had significantly higher odds to achieve (near) complete response, compared to the stroma‐high group (OR 2.85, 95% CI 1.32–6.15, *p* = 0.008). Multivariable analysis was conducted with adjustment for age, luminal subtype, morphology (ductal and lobular), clinical T‐stage and clinical N‐stage, and included complete data of 195 cases. The stroma‐low group had more than threefold higher odds to achieve (near) complete response compared to the stroma‐high group (OR 3.70, 95% CI 1.54–8.90, *p* = 0.003). The other variables were not significantly associated with the pathological outcome in the breast (Table S1).

The stroma‐low cases which showed no response after NET (*n* = 20) predominantly included patients with Grades 1–2 tumors (*n* = 5, 25% and *n* = 14, 70% respectively). In contrast, those with (near) pCR (*n* = 18) in the stroma‐low group more often had a Grade 3 tumor (*n* = 6, 33%), compared to Grade 1 (*n* = 3, 16.7%). Stroma‐high patients who did not respond to NET (*n* = 37) often had Grade 1–2 tumors as well (*n* = 9, 24.3% and *n* = 22, 59.5% respectively). Stroma‐high cases with a (near) pCR (*n* = 13) had similar numbers of Grade 1 (*n* = 3, 23.1%), Grade 2 (*n* = 7, 53.8%) and Grade 3 (23.1%) tumors. In the 153 patients with a PR+ tumor, 56 (36.6%) had a stroma‐low tumor and 97 (63.4%) a stroma‐high tumor. In the stroma‐low group, 5 (8.9%) patients achieved pCR in the breast after NET, compared to 3 (3.1%) in the stroma‐high group. A total of 13 (23.2%) stroma‐low patients achieved (near) pCR, versus 12 (12.4%) in the stroma‐high group (*p* = 0.081).

#### 3.2.2 Response of the Axilla After Neoadjuvant Therapy

In the entire cohort, 120 women had cN+ breast cancer, of whom data on the axillary response was available in 117 patients. Among them, 44 (37.6%) had a stroma‐low tumor, and 73 (62.4%) had a stroma‐high tumor.

After NET, pCR in the axilla was observed in 7 (15.9%) patients in the stroma‐low group, versus 3 (4.1%) in the stroma‐high group (*p* = 0.027).

Univariable logistic regression analysis showed that patients with stroma‐low breast cancer had significantly higher odds to reach complete response of the axilla after NET, versus the stroma‐high breast cancer patients (OR 4.41, 95% CI 1.08–18.08, *p* = 0.039).

In multivariable analysis in the cN+ cases, adjustment was performed for age, luminal subtype, morphology (ductal and lobular), and T‐stage (categories T1, T2, and T3–T4), including complete data on 112 cases. Higher odds were found to achieve response in the axilla after NET in the stroma‐low group, compared to the stroma‐high group, but this difference did not remain statistically significant (OR 6.31, 95% CI 0.93–43.10, *p* = 0.060).

#### 3.2.3 Accuracy of the TSR

The accuracy of the TSR was assessed by observing how often the expected pathological outcome based on stromal content (low/high) was consistent with the observed pathological outcomes.

In the stroma‐low group, it was hypothesized that most patients would have a partial or (near) complete response. In the stroma‐high group, the hypothesis was that the majority would have no response or a partial response at maximum.

There were 81 tumors scored as stroma‐low. In 20 (24.7%) cases there was no response in the breast achieved, 42 (51.9%) cases had a partial response, and 19 (23.5%) a (near) pCR. High stromal content was observed in 134 tumors. In 37 (27.6%) there was no response reported, in 84 (62.7%) partial response, and in 13 (9.7%) tumors (near) pCR was achieved. Altogether, the TSR correctly predicted the response of the tumor after NET in 140 (65.1%) out of 215 patients.

### 3.3 Magnetic Resonance Imaging and Pathological Response

#### 3.3.1 Accuracy of MRI

An MRI both at baseline (before the start of NET) and preoperative (after NET, before surgery) was performed in 134 (64.4%) unique patients, comprising complete data regarding MRI and pathological response of 137 tumors (including bilateral). The *κ* of radiological response (MRI) versus pathological response was 0.36. MRI findings were consistent with the pathological response in 87 (63.5%) of the 137 cases, which included 10 patients with no radiological and pathological response, 57 with partial response, and 20 with (near) complete response (Table 3).

**TABLE 3:** Radiological response of the breast measured by MRI versus actual pathological response.

Radiological response	All (*n* = 137, 100%)	No pathological response (*n* = 28, 100%)	Partial response (*n* = 85, 100%)	(Near) complete response (*n* = 24, 100%)
Radiological response				
Progression	4 (2.9)	3 (10.7)	1 (1.2)	0 (0.0)
No response	17 (12.4)	7 (25.0)	10 (11.8)	0 (0.0)
Partial	77 (56.2)	16 (57.1)	57 (67.1)	4 (16.7)
Complete	39 (28.5)	2 (7.1)	17 (20.0)	20 (83.3)

Underestimation of the MRI was defined as either partial response on the MRI or no pathological response, or as complete response on the MRI and no/partial pathological response. In 35 (25.5%) cases, MRI underestimated the presence of residual (pathological) disease. Among these cases, a partial radiological response was reported in 16 cases, but no pathological response was observed after surgery, and in 19 cases a complete radiological response was reported, but residual disease was present in resection material (Figure S1).

Overestimation of residual disease was defined as progression/no response on MRI, but partial or (near) complete response, or as partial radiological response and (near) pCR. Overestimation was observed in 15 (10.9%) cases: in 10 cases there was no radiological response described, but partial pathological response; in 1 case there was progression observed on MRI, but partial pathological response; and in 4 cases a partial response was described on MRI, but (near) pCR was reported.

#### 3.3.2 Predictive Value of the MRI in Stroma‐Low and Stroma‐High Patients

Considering MRI alone, radiological response was concordant with pathology in 87 (63.5%) cases, which included 33 (37.9%) stroma‐low and 54 (62.1%) stroma‐high tumors. Among the 16 stroma‐low cases in which the MRI showed a complete radiological response, a (near) pCR was observed in 12 (75%) cases (Table S2). In the stroma‐high group, this was the case for 8 (34.8%) of the 23 patients in which a rCR was described.

## 4 Discussion

In this retrospective cohort study, we assessed the predictive value of the TSR scored on diagnostic biopsies in HR+/HER2− breast cancer patients treated with neoadjuvant endocrine therapy, along with the radiological response versus pathological response. Identifying which patients are most likely to benefit from the neoadjuvant approach remains a clinical challenge, despite its potential advantages, such as downstaging of the tumor and axilla [24]. Although NET has historically been reserved for older patients and those with comorbidities, recent trends show an increase in the administration of NET, including younger women and fitter patients with HR+/HER2− breast cancer [25, 26, 27]. These developments underscore the importance of further research into biomarkers, such as the TSR, to better select patients who may derive the most benefit from NET.

In this study, the potential of the TSR in aiding decision‐making is demonstrated, with almost 1 out of 4 women with a stroma‐low tumor achieving (near) pCR, compared to 1 out of 10 in the stroma‐high group, highlighting a clinically relevant difference.

### 4.1 Tumor‐Stroma Ratio

The TSR was scored on 215 invasive tumors, of 208 unique patients. We found that women with stroma‐low breast cancer significantly more often reached (near) pCR of the breast after NET than those in the stroma‐high group (23.5% vs. 9.7% respectively, *p* = 0.006). There was more than a twofold increase in the odds of achieving (near) pCR after NET in the stroma‐low group, compared to the stroma‐high group. Low stromal content remained an independent favorable prognostic factor for achieving (near) pCR in multivariable analysis, after adjusting for age, luminal subtype (including grade), morphology, and clinical T‐ and N‐stage. This finding is in line with earlier research of our group, which showed that patients with a stroma‐low tumor had a better pathological response after neoadjuvant chemotherapy, compared to the stroma‐high group [18]. Other studies, in breast cancer and esophageal cancer, also showed better response rates to respectively neoadjuvant therapy and chemoradiotherapy in patients with a stroma‐low tumor [28, 29].

A hypothesis of the worse response rates in the stroma‐high group is that the stroma could function as a protective barrier surrounding the tumor, which may reduce the efficacy of administered therapy. Another possibility is the occurrence of more high grade tumors in the stroma‐low group, as found in Hagenaars et al. and in this current study, supported by the theory that high grade is correlated with higher proliferation rates and therefore better response to treatment [18, 30]. These findings suggest that tumor grade may partly explain the heterogeneity in treatment response observed across the stroma‐low and stroma‐high groups: in the stroma‐low group, women with Grade 1 tumors were more likely to be poor responders and those with Grade 3 tumors better responders, while in the stroma‐high group, a subset of Grade 3 tumors showed an unexpectedly favorable response. A study of Lips et al. demonstrated that histological grade had the best predictive value for response to chemotherapy, with Grade 3 tumors having a higher odds to achieve pCR, compared to Grades 1 and 2 tumors [31]. This correlation was also described in other studies, mainly regarding chemotherapy, but was also observed in our current study regarding NET [30, 32, 33].

In the axilla, stroma‐low patients with cN+ breast cancer also demonstrated better response to NET compared to the stroma‐high patients. Again, an increased odds ratio to achieve response was observed in univariable analysis among those with stroma‐low breast cancer. After adjustment for age, luminal subtype, morphology, and clinical T‐stage, the significance observed in univariable analysis regarding response of the tumor and axilla correlated to the TSR did not remain in multivariable analysis. This might be due to the relatively small sample size or potential correlations between covariates.

In contrast to previous studies reporting better response rates in luminal B patients, we did not find a difference in response of the breast between the luminal A‐like and luminal B‐like subtypes [34, 35]. It is hypothesized that they demonstrate better response rates due to higher proliferation in luminal B cancer, but these studies have mainly focused on the response to chemotherapy and not to endocrine therapy, which we focused on in this study [34, 35]. Conversely, the luminal A subtype is generally regarded as the most endocrine‐sensitive [36]. It is therefore possible that the relatively small subgroup sizes in our study have masked this effect.

### 4.2 Magnetic Resonance Imaging

The MRI remains the gold standard to evaluate response of neoadjuvant therapy before surgery. However, studies on MRI in NET are limited and show underestimation of the tumor size, which may increase the risk of positive surgical margins [12]. Therefore, we examined both the TSR and MRI in this study to explore their respective roles in predicting and assessing treatment response following NET. The accuracy of the TSR and MRI was assessed by analyzing how often a correct prediction and/or observation was seen, corresponding with pathological outcomes. A discouraging Cohen's *κ* of only 0.36 was found between radiological and pathological response, which indicates fair agreement. However, we did find that the accuracy of MRI in correctly predicting radiological complete response was higher in the stroma‐low group (75%), compared to the stroma‐high group (35%). The higher accuracy in the stroma‐low group might be due to better visualization of therapy‐induced tumor regression, whereas in the stroma‐high group extensive stromal and/or fibrotic tissue may limit the ability of the MRI to distinguish residual tumor cells from treatment‐related changes such as fibrosis. This observation may help provide more insight at the start of NET into how accurately the MRI can predict (near) pCR. However, the numbers in this study reporting this finding were small, so validation in a larger cohort is needed. A direct comparison between the TSR and MRI is not possible since both modalities focus on different aspects: the TSR reflects tumor biology and the composition of the TME, whereas the MRI reflects radiological changes after therapy. Moreover, the TSR is scored before start of NET, while an MRI is performed during therapy in a longitudinal setting to detect changes.

Less underestimation of response was observed in our study, compared to earlier studies on MRI and NET (25.5% vs. 68.6%–77% respectively), but residual disease was still underestimated in approximately 1 out of 4 women [12, 37]. Underestimation of tumor poses a clinical risk, as it may lead to an increase of positive surgical margins due to more residual disease being present than observed on the MRI. This is particularly the case in patients treated with NET, as a result of fragmented regression (cookie‐crumble effect), rather than a concentric shrinkage, as is observed more in patients treated with NACT [37]. Furthermore, our study evaluated the occurrence of response, instead of evaluating clinical and pathological tumor size. It is therefore possible that the extent of underestimation in our study would be greater had we assessed clinical versus pathological size on the MRI instead.

### 4.3 Definition of Response

A methodological challenge in this study was analyzing the predictive value of the TSR. In the Pearson chi‐square analysis the predictive value of the TSR was considered ‘correct’ when stroma‐low tumors demonstrated a (near) pCR, and stroma‐high tumors in case of no response or a partial response at best. As a consequence, a considerable number of partial responders were grouped with the non‐responders, which likely led to an underestimation of the TSR's predictive performance, particularly in the stroma‐low group. Therefore, the actual accuracy of the TSR in the stroma‐low group is most likely higher, since some degree of treatment response would be expected in this group (as presented in Figure S1). This limitation primarily lies in the categorization of partial response, which remains challenging to define. In this current study, patients classified as partial responders had 10%–90% tumor rest after NET, which represents a broad range. Greater consistency could be achieved by applying existing classification systems, such as the MP and RCB, in a more uniform manner, to limit the variability in the partial response group.

### 4.4 Pathological Complete Response in HR+/HER2− Patients

Another challenge is the low rate of pCR observed in HR+/HER2− breast cancer patients [38]. Earlier studies mostly report pCR rates following NACT, and in HR+/HER2− patients, the frequency of pCR differs between those receiving NACT and those receiving NET. Studies have reported pCR rates ranging from 6.3% to 19.3% in HR+/HER2− patients treated with NACT, whereas rates after NET are around 0%–3% [39, 40, 41]. In our study, a pCR rate of 6.1% was observed after NET, which is in line with these earlier studies. The low pCR rates after NET make it challenging to identify a robust and reliable predictive marker in HR+/HER2− patients treated with NET. Nonetheless, nearly 1 out of 4 women with a stroma‐low tumor achieved (near) pCR, highlighting a clinically meaningful difference compared to the lower response rates in the stroma‐high group.

Although the median duration of NET in this study was within the guideline‐recommended 6–9 months, there was considerable variation, with timing individualized based on tumor response, patient‐ and tumor‐specific factors, logistics, and patient preferences. Primarily, it was used to enable breast‐ and/or axilla‐conserving surgery in line with the Dutch guideline [6, 41]. Other factors that influenced the duration or intended aim of NET included delay of surgery due to additional procedures or concurrent malignancies, disease progression requiring earlier surgery, COVID‐related scheduling limitations, or patient preference (such as postponing surgery for vacation).

### 4.5 Upfront Surgery Versus Neoadjuvant Endocrine Therapy

An earlier observational cohort study of our research group in 7809 stage II‐III, HR+/HER2− breast cancer patients demonstrated that 51.8% of the patients had directly undergone surgery (PS), whereas the other patients received neoadjuvant therapy [41]. In the PS patients, 84% received endocrine therapy in the adjuvant setting. A potential approach is to shift part of the current standard of care, namely 5‐year endocrine therapy in the adjuvant setting, to the neoadjuvant setting, thereby increasing the feasibility of less invasive surgery on the breast and axilla.

The potential lies particularly in this group of patients, who currently undergo upfront surgery. The TSR could play a role in this process of clinical‐decision making. For example: if patients with stage II‐III, HR+/HER2− patients, who are set to receive 5 years of endocrine therapy regardless, are informed that 1 in 4 women with a stroma‐low tumor achieves a (near) pCR, more may opt for NET as the initial treatment, instead of upfront surgery. This, in turn, could increase the likelihood of undergoing breast‐conserving surgery rather than a mastectomy. In this scenario, there is no change in the type of therapy, but the timing is adjusted: shifting from an adjuvant approach to a (partially) neoadjuvant setting to benefit more from the advantages of neoadjuvant therapy.

This theory is particularly accessible by the relatively easy and low in cost method of determining the TSR. It is scored on the diagnostic biopsy using an H&E slide, which is already routinely assessed, with no requirement for additional immunohistochemical (IHC) staining. The process of scoring the TSR is especially easier when compared to other markers, such as the Ki67, which requires additional IHC staining, with variable cut‐off values and interobserver variability [42, 43]. The interobserver agreement of the TSR is relatively high, ranging from 0.68 to 0.85, and is evaluated using a standardized protocol [20, 44].

A limitation of this study is the relatively small sample size, which is partly due to the emerging, but previously limited use of NET and the (previous) tendency to administer it primarily to older women. This made subgroup analyses, such as in *N*+ patients, difficult. Another limitation is the different use to describe pathological response. The different use of the Miller‐Payne grading system and Residual Cancer Burden posed a challenge to obtain a single, uniform outcome measure. During the reclassification of the response groups into three categories, misclassification may have occurred due to the use of these different grading systems, for example in cases where response rates were less clearly defined. Another potential limitation is combining patients from the NEOLBC trial with standard of care patients, which could introduce bias from cohort‐specific selection or workflow differences.

Nevertheless, key aspects of treatment, imaging, and follow‐up were largely standardized (according to Dutch guidelines), and eligibility criteria were comparable, supporting that the observed association between TSR and response likely reflects a biological effect rather than cohort‐specific factors. Finally, a limitation is the retrospective setting, which resulted in limited availability of data on MRI response rates. This is noteworthy, as MRI data were available in only 64% of patients at both baseline and before surgery, which may have introduced selection bias and should be considered when interpreting the MRI‐related findings. Nevertheless, these results highlight the need for more standardized and complete imaging assessment to further improve evaluation of neoadjuvant therapy response. The availability of Ki67 at both baseline and in the resection tissue was also limited, which could have been used to assess the change in Ki67 during treatment and compare the TSR to other biomarkers.

Future studies should focus on validating the TSR in a larger cohort of patients treated with neoadjuvant therapy (both NACT and NET). A prospective study, for example, could incorporate complete data on Ki67 at baseline and in the resection material, along with exact data on the tumor size on the MRI and the pathological size. Adding the TSR to existing tools, such as PREDICT, could improve the model further, as has been demonstrated in an earlier study of our group (accepted for publication) [45]. Future research could also focus on the combination of the TSR with other biomarkers, such as Ki67 and the promising tumor‐infiltrating lymphocytes, which might also be interesting to evaluate [44], as they could potentially complement and strengthen each other's predictive value.

## 5 Conclusion

In this study, which included women with HR+/HER2− breast cancer, nearly 1 out of 4 patients with a stroma‐low tumor achieved (near) complete response of the breast after treatment with NET. While MRI remains the current gold standard for preoperative treatment assessment, underestimation of residual disease occurred in a quarter of the women. The TSR promises to have additional value to MRI in patients treated with NET.

## Author Contributions

**Layla Andour:** conceptualization, methodology, investigation, formal analysis, visualization, writing – original draft, writing – review and editing, data curation. **Sophie C. Hagenaars:** investigation, writing – review and editing. **Anne Florine de Groot:** writing – review and editing, data curation. **Elly M. M. Krol‐Warmerdam:** writing – review and editing, data curation. **Judith R. Kroep:** writing – review and editing. **Hans Marten Hazelbag:** writing – review and editing, investigation. **Gerrit‐Jan Liefers:** writing – review and editing. **Marieke E. Straver:** writing – review and editing, visualization. **Wilma E. Mesker:** visualization, writing – review and editing, supervision, funding acquisition, conceptualization.

## Funding

This research was financially supported by the Bollenstreekfonds, Lisse, Netherlands.

## Ethics Statement

The current study was conducted in accordance with the Declaration of Helsinki and with approval of the Ethical Committee of the Leiden University Medical Center, in the Netherlands. Data for this study were obtained from the NEOLBC trial and routine clinical care. The Netherlands Comprehensive Cancer Organization (IKNL), which collects and processes data in accordance with national data protection regulations, provided an updated list of patients receiving neoadjuvant endocrine therapy in the participating hospitals. Patients participating in the NEOLBC trial provided informed consent. Additional informed consent and ethical approval (routine clinical care) were not required due to the use of anonymized data.

## Conflicts of Interest

The authors declare no conflicts of interest.

## Supporting information


**Table S1:** Multivariable Cox Regression analysis regarding achievement of (near) complete pathological response after neoadjuvant endocrine therapy.
**Table S2:** Predicted response of MRI and TSR compared actual pathological response.
**Figure S1:** Outcomes of preoperative Magnetic Resonance Imaging (MRI) and stromal content, related to pathological outcomes.

## Data Availability

The data that support the findings of this study are available from the corresponding author upon reasonable request.
