# Supplementary material for: The Predictive Value of the Tumor‐Stroma Ratio for Neoadjuvant Endocrine Therapy in Hormone Receptor‐Positive Breast Cancer
Source: Int J Cancer. 2026 Apr 10;159(4):1056–67. doi: 10.1002/ijc.70490 (PMC13284621; doi:10.1002/ijc.70490)
Supplement: Supplementary file 1 — Table S1: Multivariable Cox Regression analysis regarding achievement of (near) complete pathological response after neoadjuvant endocrine therapy. Table S2: Predicted response of MRI and TSR compared actual pathological response. Figure S1: Outcomes of preoperative Magnetic Resonance Imaging (MRI) and stromal content, related to pathological outcomes. [file IJC-159-1056-s001.pdf]

Supplementary Material for

The Predictive Value of the Tumor-Stroma Ratio for Neoadjuvant Endocrine Therapy in Hormone Receptor-Positive Breast Cancer

Layla Andour, Sophie C. Hagenaars, Anne Florine de Groot, Elly M.M. Krol-Warmerdam, Judith R. Kroep, Hans Marten Hazelbag, Vincent T.H.B.M. Smit, Marieke E. Straver, Gerrit-Jan Liefers, Wilma E. Mesker

Table of Contents

Page 2: Supplementary Table 1: Multivariable Cox Regression analysis regarding achievement of (near) complete pathological response after neoadjuvant endocrine therapy
Page 3: Supplementary Table 2: Predicted response of MRI and TSR compared actual pathological response
Page 4: Supplementary Figure 1: Outcomes of preoperative Magnetic Resonance Imaging (MRI) and stromal content, related to pathological outcomes

Supplementary Table 1: Multivariable Cox Regression analysis regarding achievement of (near) complete pathological response after neoadjuvant endocrine therapy

<u>**(Near) complete response**</u>	**OR**	**95% CI**	**p-value**
**Variables**			
**TSR**			
Stroma-low	3.70	1.55-8.90	0.003
Stroma-high	1		
**Age**	1.03	0.99-1.07	0.170
**Luminal subtype**			
Luminal A-like	1		
Luminal B-like	0.89	0.37-2.13	0.793
**Morphology**			
Ductal	1		
Lobular	1.49	0.57-3.90	0.414
**Clinical T-stage**			
I	1		
II	0.45	0.16-1.30	0.140
III	0.70	0.17-2.91	0.625
IV	1.18	0.31-4.55	0.809
**Clinical N-stage**			
N0	1		
N+	2.05	0.82-5.11	0.126

n = 10 patients excluded from multivariable analysis due to missing data or small groups

Supplementary Table 2: Predicted response of MRI and TSR compared actual pathological response

**Stromal content and radiological response combined**	**All (n = 137, 100%)**	**No response (n = 28, 100%)**	**Partial response (n = 85, 100%)**	**(Near) complete response (n = 24 , 100%)**
TSR and MRI				
Stroma-low, MRI no response	5 (3.6)	1 (3.6)	4 (4.7)	0 (0)
Stroma-low, MRI partial response	31 (22.6)	9 (32.1)	20 (23.5)	2 (8.3)
Stroma low, MRI complete response	16 (11.7)	0 (0)	4 (4.7)	12 (50.0)
Stroma-high, MRI no response	16 (11.7)	9 (32.1)	7 (8.2)	0 (0)
Stroma-high, MRI partial response	46 (33.6)	7 (25.0)	37 (43.5)	2 (8.3)
Stroma-high, MRI complete response	23 (16.8)	2 (7.1)	13 (15.3)	8 (33.3)

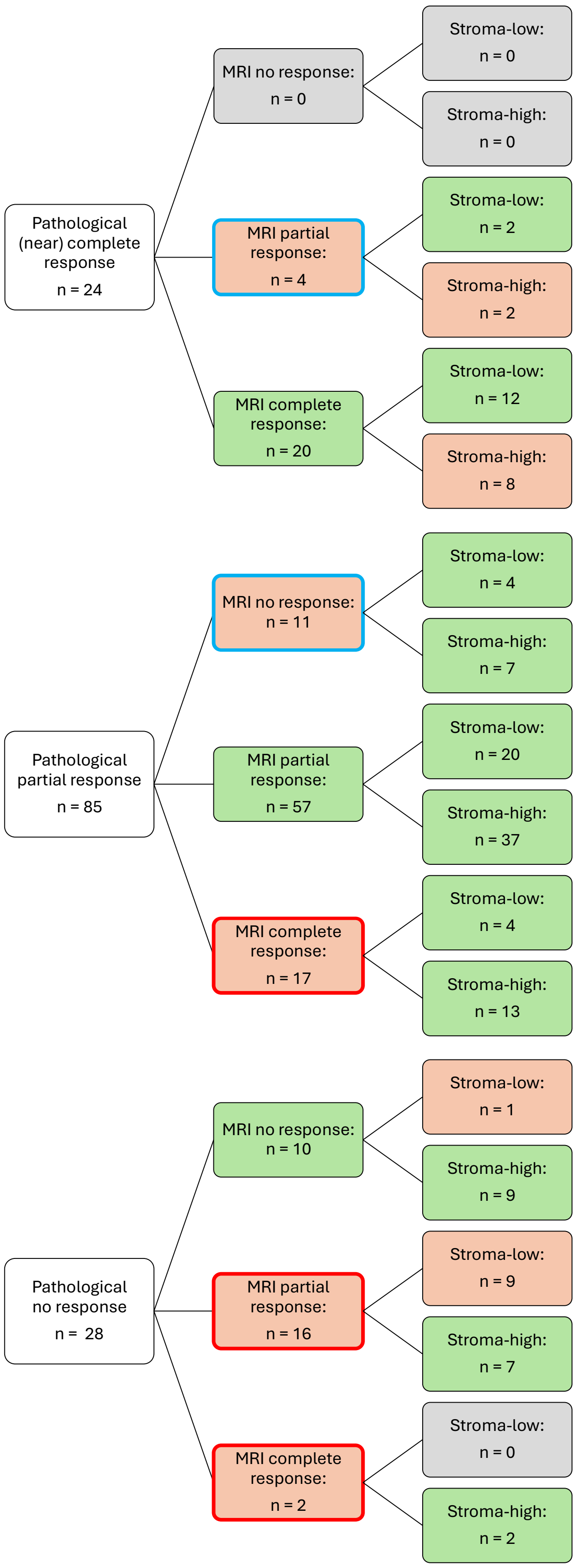

Supplementary Figure 1: Outcomes of preoperative Magnetic Resonance Imaging (MRI) and stromal content, related to pathological outcomes

Green: correctly classified radiological response and/or stromal content related to pathological response.
Orange: misclassified radiological response and/or stromal content related to pathological response; orange-blue = overestimation of residual disease by MRI; orange-red = underestimation of residual disease by MRI.
Grey: not applicable (n = 0).
